# The Amendments to the Maryland State Dental Law

**Published:** 1890-02

**Authors:** 


THE AMENDMENTS TO THE MARYLAND STATE DENTAL LAW.—The following amendments to the present State Law regulating the practice of dentistry in Maryland, are now before the Legislature: Providing for a board of examiners of persons who propose to practice dentistry in this State. Five dentists shall constitute the board—three from Baltimore city, one from the Eastern and one from the Western Shore. The members of said board shall be appointed by the Governor, each from two names of dentists to be presented to him by a mass-meeting of the registered dentists. Dentists who have engaged in practice in this State prior to March 31, 1884, and who shall desire to continue, shall register with said board of examiners. Any person who has graduated and holds a diploma may present the same to the board of examiners for approval. All other persons who desire to practice dentistry in this state shall be examined by the board with reference to their skill and knowledge in dental surgery. The sum of $5

shall be charged for every certificate issued by the board, and $10 from every person who shall appear for examination. Fines of $50 to $300 and imprisonment for six months are provided for violations of these provisions. One member of the board may, upon proper evidence, grant a temporary certificate to any applicant. Nothing in this bill shall be so construed as to interfere with the rights and privileges of resident physicians and surgeons. The persons who constitute the present board of examiners shall continue to act for the residue of their terms, and nothing in it shall be construed to render it necessary for any person who holds a certificate from the present board to again register.

